# Interactive 3D Objects Enhance Scientific Communication of Structural Data

**DOI:** 10.1002/cbic.202500036

**Published:** 2025-02-20

**Authors:** Daniel Mokos, Bastian Daniel

**Affiliations:** ^1^ Department of Structural Biology Institute of Molecular Biosciences University of Graz Humboldtstraße 50 8010 Graz Austria; ^2^ BioTechMed Graz Austria

**Keywords:** Structural biology, Bioinformatics, Structure elucidation, Communication, Structure representation

## Abstract

In scientific communication about three‐dimensional structures, creating two‐dimensional representations is standard practice. These representations often suffer from the drawback of losing potential information due to dimensionality reduction. Several options exist to present, share and publish 3D figures, however based on recent publications they are not widely utilized. Here we present simple ways to preserve the three‐dimensionality of the structure by the creation of a custom‐made model in GLTF format that is generated in the same workflow as the conventional figures. They can be published alongside a given manuscript with minimal additional effort to the authors, but a huge impact on the communicative power of the manuscript concerning the three‐dimensional features of the reported structures. The scripts we adapted and published for this purpose open up new possibilities for the illustrator and allow the viewer to access the full three‐dimensionality of the published structure. In future, this can simplify the publication process of protein structures or other models and be a valuable tool for scientific communication in digital or printed form.

## Graphical abstract

In this perspective, the enhancement of scientific communication through interactive 3D models is presented. In contrast to 2D figures, the use of augmented reality and GLTF files can provide readers with a more comprehensive perception of structural data, preserving the three‐dimensional complexity of published structures. This method has the potential to have a transformative effect on the authors’ efforts to present complex data and improve readers comprehension of molecular interactions at the same time.

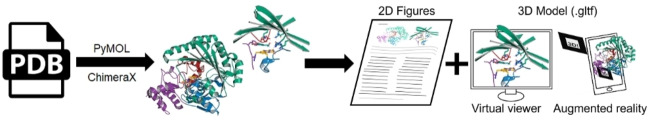

## Introduction

*“To understand biology, one must think in a language of three dimensions, a language of shape and form… in biology, especially at the cellular and molecular levels, nearly all activity depends ultimately upon form, upon physical structure…”* John M. Barry.^[1]^

In order to master this language, the ability to comprehend biological structures in the spatial realm is crucial. Unfortunately, the amount of spatial information pertaining to biological structures in the current literature is not as comprehensive as it could be.

Structural biology aims for the elucidation of the three‐dimensional structure of biological macromolecules such as proteins, nucleic acids or complete viruses.^[2]^ The knowledge of the spatial arrangement of the respective molecules at atomic level allows us to understand the molecular interactions that govern their functions. It allows fundamental insights into shape, size and function of biomolecules that ultimately can be used in drug discovery, design of biomolecules and the elucidation of disease mechanisms. Representations of macromolecular structures are therefore a key tool in scientific communication, as they support collaboration, construction of new knowledge for didactical purposes, and in general, support scientists’ professional activities.^[3]^ Due to the high complexity of the of macromolecules, simplified representations of the respective structures are necessary. Based on recent publications, the general practice is to create 2D static images using a visualization software like PyMOL^[4]^ or ChimeraX,^[5]^ in which the important features of a structure are highlighted. The loss of the third dimension, however, leads to limitations in the amount of spatial information, that can be conveyed through figures.

The need for 3D figures has been an important topic for both scientific communications as‐well‐as education.^[6]^ There are already several options to publish or present 3D structures like FirstGlance in Jmol, enhanced figures in Acta Crystallographica, or the more recent 3DMol.js, Mol*, 3dRS and MoleculARweb.[^7^, ^8^, ^9^, ^10^, ^11^, ^12^] The development of web‐based programming languages has facilitated the establishment of JavaScript and webGL applications that can host macromolecular structures and can be shared alongside publications or used as a visualization tool. In recent years, with the rise of virtual‐ and augmented‐reality, new tools appeared to present structural data, mostly for educational purposes.[^12^, ^13^, ^14^] The above‐mentioned applications are built to be independent from other software, and handle structure files directly. Due to this nature, the preparation of the 3D figure is separated from the conventional figures, they require significant efforts from the authors, and the tools end up severely underutilized in publications. The viewing experience is also affected by this, as the viewers need to load the entire application, the structure file, and the settings of the illustrator, which can lead to elongated loading time or lag. Although the advantage of 3D objects in scientific communications has been described thoroughly and there are sophisticated tools available, none of them are used as in the 10 most recent papers in Nature, JBC, ACS and Acta Crystallographica covering structural data (date 20. 12. 2024)

To overcome these limitations, we set out to create an accessible, lightweight solution based on the same workflow that is used to generate a conventional 2D figure. A three‐dimensional object in GLTF format can be created by the latest PyMOL and ChimeraX versions the same way an image is prepared, with the same customizability. Utilizing existing web‐based tools AR.js and < model‐viewer> [^15^, ^16^] these models can be hosted on small and intuitive websites. Journals currently host high resolution figures, videos, and PowerPoint slides alongside the actual manuscript, and in the ideal case, the 3D objects could be hosted by the journal as well. The implementation of this interactive tool into publications or presentations allows the audience to explore the three dimensional properties of the corresponding macromolecule prepared by the expert quickly and effortlessly. To the reader accessing this custom‐made 3D model is just as effortless as viewing a high‐resolution picture instead of the web‐version.

### Examples

**Figure 1:**
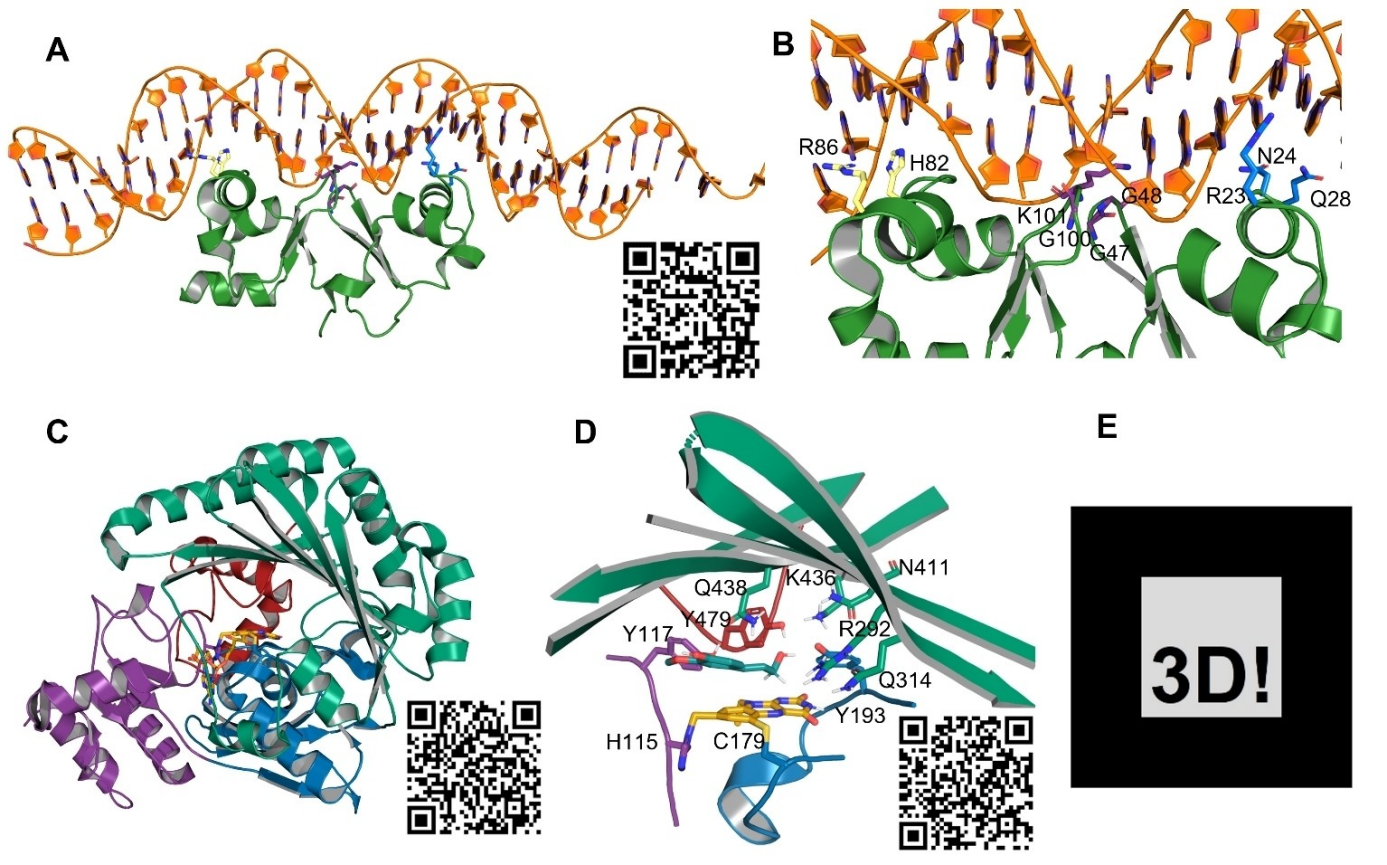
**A**: Overall topology of TraN in complex with DNA, **B**: Specific interactions between TraN and the DNA double strand. 3D scene: https://mokosdaniel.github.io/TraN_mv/ **C**: Overall topology with respect to the subdomain organization of the berberine bridge‐enzyme‐like (BBE‐like) protein *At*BBE‐like 15. 3D scene: https://mokosdaniel.github.io/BBE15overview_mv/ **D**: active site of *At*BBE‐15 with the substrate coniferyl alcohol and the active site forming residues. 3D scene: https://mokosdaniel.github.io/BBE15bind_mv/ **E**: Marker image for the Augmented reality scenes accessible through the QR codes in the respective panels.

The methodology we describe here was previously utilized to present substrate and product complexes of the *Sphingomonas sp*. Lipase SpL that were published elsewhere.^[17]^ To further elaborate the advantages of this method, we have chosen a limited set of examples to demonstrate how 3D objects can enhance scientific communication. In Figure 1 several examples of macromolecules are depicted with links to the respective three‐dimensional objects in the figure legend to demonstrate, how the viewer can benefit from the supplementation of a 3D object and how that facilitate the publishing process for the illustrator as the 3D object compensates for the loss of one dimension in the creation of the figure.

Our first example is TraN, that plays a role in the type IV secretion system of bacteria, that facilitates gene transfer during conjugation.[^18^, ^19^] TraN is a small cytosolic protein that acts as a repressor of the pIP501‐encoded conjugative transfer system in *Enterococcus bacteria*. Its deletion results in upregulation of transfer factors, leading to highly enhanced conjugative transfer. The exact sequence of its binding motif has been defined and the protein was crystallized depicted in complex with DNA in Figure 1 panel A and B. Kohler *et al*. describe a complex set of interactions, that to some extent also rely on the shape complementarity of the DNA and TraN. There are three sets of residues that are interacting with the DNA, Arg86 and His82 are depicted in yellow and bind in the major groove, an interaction with the minor groove is established via Lys101, Gly100 and Gly47 and additionally an interaction is formed between Arg23, Asn24 and Gln28. Due to the high complexity of the interactions, it is not possible to represent all of them satisfactorily in one illustration. With a 3D object, the viewer has the possibility to work through all the views that are individually necessary to grasp the three‐dimensional relationships of the interaction between TraN and the DNA. Also the shape complemenarity of TraN to the DNA is clear from the 3D object, while this is aspect is lost in the two‐dimensional figure. Additionally, the 3D object is of high educational value. The concept of the DNA double helix, the minor and major groove and the stacking of the nucleotides can be anticipated to be standard knowledge in life sciences. Nevertheless, the number of people that actually have seen the structure of the DNA derived from primary data as it is presented here is still limited.

As second example we have chosen the flavin‐dependent monolignol oxidoreductase *At*BBE‐like 15.^[20]^ The enzyme can be divided in the flavin‐binding modules depicted in blue, purple and red, that are opposed by a substrate binding domain depicted in green.^[21]^ The flavin‐binding modules mainly interact with the ribityl residue and the adenine, while the isoalloxazine ring, the catalytically active moiety of the bi‐covalently attached cofactor (depicted in orange), is localized at the interface of all domains and oriented towards the active site. The color code introduced to the 3D‐object makes it easy to identify the individual subdomains, while the possibility to rotate the respective object gives the viewer the possibility to explore each interface individually and also their orientation towards the flavin cofactor. Additionally, we focused on the active site of *At*BBE‐like 15, that was previously described by Messenlehner *et al*. in more detail.^[22]^ We postulate a complex network of polar residues in the active site to be involved in the binding and activation of the alcoholic substrate, the stabilization of the respective alkoxide intermediate and to catalyze the hydride transfer to form the respective aldehyde. We consider the quality of the scientific communication that is possible with the 3D object unmatched by any of the two‐dimensional figures that were published on that matter before. We postulate Tyr479 and Tyr193 to concertedly deprotonate the substrate while their catalytic properties are being regulated via Lys436. In Panel D of Figure 1 we have recreated the depiction of this enzyme substrate complex. The three‐dimensional relationships between the three residues and the substrate can easily be explored by the viewer using the 3D object. In addition, the position of the substrate can also be related to other important interaction partners such as Gln438 and Tyr117 or Arg282 and Gln314. Due to the complexity of the structure and the numerous interaction partners the communicative power of the 3D object in comparison with the 2D figure is much higher, although they originate from the same PyMOL session. *I. e*. once the authors have elaborated the structural features that are to be depicted for the creation of a figure, the additional export of an 3D object can boost the accessibility of the respective information to the viewer. Furthermore, the color code in combination with panel C shows how the different subdomains are involved in the attachment of the cofactor and which residues they contribute to the active site. This makes the 3D object a valuable source of information that can be used by the viewer according to their own level of interest and pace.

### Methods

Currently these steps must be done by the authors. If the GLTF files would be hosted by the journal, it would be just another file the authors have to upload in the submission process. We strongly encourage all editors make this possible.

We describe here two types of web‐based applications for the visualization of a 3D model; a virtual model viewer and an augmented reality viewer. A detailed tutorial is available alongside the code for both (https://github.com/MokosDaniel/modelviewer, https://github.com/MokosDaniel/ARPymol). Any GLTF model can be used with these tools. Typically, they are created using PyMOL or ChimeraX. To create a 3D‐model of your structure of interest, load and customize the model in PyMOL 2.4 or above. Set up the scene exactly as it is to be presented in 3D, highlight the interesting features by coloring them, or representing them in a different way. To generate the 3D object, use the command:′ save filename.gltf ′, or select ′ File – Export image – GLTF… ′ and save the file. It is also possible to use ChimeraX to generate the model, with the same steps. In ChimeraX commands can be used to set the quality of the models which can help reduce the file size significantly. Models can be saved in File – Save… as the file type “.glb” or with the command: ′ save filename.glb ′^[23]^. The file size of a 3D model depends on other parameters then a 2D‐figure. In the tutorial is described how to avoid the creation of unnecessary big files by for example avoiding mesh representation, and minimizing the number of sticks, balls and lines. We recommend surfaces and cartoon representation for a reasonable file size and for the ease of understanding important residues and molecules should be represented as sticks. The current approach requires a GitHub repository and the file‐size limit for GitHub is 25 MB. Additionally larger files result in a slower user experience. Transparency and labels will not be applied in the model, but measurements will be visible, without values. The model can be further customized in any 3D sculpting program (e. g., Blender), to introduce transparent details, customize materials, add labels, and even animate the scene, as exported GLTF files can carry all this information. The 3D scene can be hosted on an HTML website. For beginners the easiest way to do this is with GitHub Pages, for experienced users, it is possible to create and host a standalone website, or to integrate it into an existing site with the code found in the example repository. Ideally, files would be hosted by the respective journal publishing the article in future.

### Augmented Reality

For printed media, especially posters, an augmented‐reality application is desirable, where the presented model is tied to a marker image, that can be viewed with devices that have cameras. This enables the author to position the model in relation to the content of the publication, and the explanation of the figures. For this purpose, every 3D scene should be a separate GitHub repository. The necessary code to evoke a model from a QR‐code and a marker (see Figure 1 panel E) is given in the example repository https://github.com/MokosDaniel/ARPymol. It will provide the detailed setup steps necessary in the Readme file. In short, these steps include uploading of the model, changing of the model's name in the code, and generating the website through GitHub Pages. Viewing the AR scene is possible by following the URL shown in the GitHub settings under Pages. The reader can access the scene on any device with a camera, by scanning the QR‐code, leading to the URL. By pointing the camera at the marker image in Figure 1 panel E will evoke the model. It is possible to enlarge or shrink and rotate the model with gestures on a touchscreen. The scene is always anchored to the marker image, meaning this has to be available to view the model. Printed markers tend to work better than ones displayed on screens.

### Virtual Model‐Viewer

A virtual scene can be more desirable for publications intended to be consumed on screens, in browsers with several tabs, where the link for the figure can be opened alongside the publication, or even be implemented directly into an html format of the article. The respective code to set the virtual scene is available at the example repository https://github.com/MokosDaniel/modelviewer, that will provide the setup steps in the Readme file. The limitations and usage described above also applies. Goddard described additional coding tips for this application.^[23]^ In this case generating a QR code can be useful to share the website, but the URL itself is also usable, as the interactivity works on all devices capable of displaying a HTML site. It is still important to use the URL of the hosted website (found in ‘Settings – Pages’), not the URL of the GitHub repository. The experience works in any environment, there is no need for a marker or any physical aid, as it is a complete virtual scene.

## Conclusions

In summary, this perspective introduces an innovative approach for scientific communication that leverages 3D objects and augmented reality to present complex three dimensional structures. The preparation of figures for scientific communication has been established for many years. Here we demonstrate how the same workflow can lead to significantly enhanced communication of structural data with very little additional effort employing augmented reality or a model viewer. The possibility to export, present and view customized models as GLTF files makes presenting and understanding spatial information far more accessible to both authors and readers. This methodology is easy to apply and offers the opportunity to broaden the way structural biology research is shared and understood in both digital and print media. With 3D objects that are carefully prepared by the authors of an article, the reader can work out the three‐dimensional relationships that are covered in the respective scientific work interactively. Even details that are difficult to depict when converting a 3D object into a two dimensional image can be preserved in this way. This means easier figure preparation for the authors, but at the same time the interactive access allows the viewer to perceive and understand more details in the images in combination with a 3D object.

## Conflict of Interests

The authors declare no conflict of interest.
